# Increasing Environmental Complexity by Providing Different Types of Litter and Perches during Early Rearing Boosts Coping Abilities in Domestic Fowl Chicks

**DOI:** 10.3390/ani12151969

**Published:** 2022-08-03

**Authors:** Franco Nicolas Nazar, Lena Skånberg, Kirste McCrea, Linda Jane Keeling

**Affiliations:** 1 Instituto de Ciencia y Tecnología de los Alimentos (ICTA), Facultad de Ciencias Exactas, Físicas y Naturales, Universidad Nacional de Córdoba (UNC), Cordoba X5016GCA, Argentina; 2 Instituto de Investigaciones Biológicas y Tecnológicas (IIByT, CONICET-UNC), Consejo Nacional de Investigaciones Científicas y Técnicas (CONICET), Cordoba X5016GCA, Argentina; 3 Department of Animal Environment and Health, Swedish University of Agricultural Sciences, SE-750 07 Uppsala, Sweden; kirste.mccrea@slu.se (K.M.); linda.keeling@slu.se (L.J.K.)

**Keywords:** adaptability, immunology, stress, laying hens, animal welfare, resilience, development, learning

## Abstract

**Simple Summary:**

The benefits of rearing chicks in complex environments rather than barren environments are well established. However, the typical rearing environments for modern laying hens are still considerably more barren than the complex forest habitat of their ancestors. This small-scale experimental study investigated whether giving chicks of white Bovans Robust the possibility to express choices between different variants of the same resource could result in them being better able to cope with challenges, as well as being better able to make the most of new opportunities. We found that chicks with access to different types of litter and perches were less fearful, less chronically stressed, and were better prepared to cope with pathogenic challenges. Furthermore, they were more successful in a repeated opportunity test, implying an improved learning ability. Overall, the results suggest that rearing laying hen chicks in an environment with access to variation in relevant resources could be a simple and feasible way to increase complexity under commercial conditions. This input could result in them being more resistant to infection and better able to adapt to novel situations later in life.

**Abstract:**

Early experience of a complex environment can improve biologically relevant traits related to coping abilities. However, the mechanisms underlying these positive effects have not been well explored. We hypothesized that giving chicks possibilities to express choices within relevant resources could be an important part of the mechanism, as well as a novel way to increase environmental complexity. In a balanced design, laying hen hatchlings of the white hybrid Bovans Robust were reared in a “single-choice” environment (single litter and perch type) or a “multi-choice” environment (four different litter and perch types). Immunological and behavioral indicators of chicks’ coping abilities were explored in this experimental study at three weeks of age. Chicks from “multi-choice” environments had shorter durations of tonic immobility, lower heterophil/lymphocyte ratios, higher natural antibody concentrations, and were more successful in gaining novel food rewards in a repeated opportunity test. These results imply that chicks having access to variation within resource types were less fearful, experienced less chronic stress, would be more able to cope with pathogenic challenges, and potentially had an improved learning ability. To conclude, the more complex environment, achieved by increasing chicks’ possibilities to choose, seemed to make chicks better prepared for potential challenges, boosting their adaptive capacities and their ability to make the most of opportunities.

## 1. Introduction

Farm animals are potentially exposed to various challenges and stressors throughout life, such as routine handling, transport, and infections [1,2,3]. Laying hens are seen as particularly sensitive to these challenges, although there are already many practices in place to protect them, especially during rearing. For example, it is a standard practice to disinfect the building between batches of birds, and many producers knock on the door before going inside a rearing stable to reduce flight responses. However, the scientific literature suggests that coping with some challenges early in life can lead to a more resilient individual. For example, research in rodents suggests that early experience of stress can improve stress coping (i.e., how individuals respond to potential stressors) later in life [4,5]. While minimizing welfare risks is of course important, one can question whether chicks might be over-protected during rearing, leading to sub-optimal development of their ability to cope with the stressors they will most likely experience later in life. In short, can we better prepare chicks by manipulating their early environment?

Experiences early in life are of pivotal importance for the development of a range of abilities. Highly relevant developmental processes occur in the first weeks of life, and this early temporal window is suggested to influence neuroendocrine components, i.e., the hypothalamic–pituitary–adrenal (HPA) axis [6], the immune system, and how these two systems interact [7]. In avian species, hatchlings encounter an entirely new environment, far from the security offered by the in ovo situation. This new environment is full of challenges, ranging from escaping the shell, to exploring this new world and resisting a series of potential pathogens [8]. According to the adaptive development plasticity theory, the environment that individuals encounter in the early stages of their development, and their genetic potential to respond to this environment, will determine their later phenotype and thereby affect their fitness [9]. Within this framework, the particular way each individual responds to challenges can reflect their current coping abilities and, at the same time, provide an indication of their future coping abilities.

The typical rearing environments for modern laying hens are considerably more barren than the complex forest habitat of their ancestors, and it has been suggested that barren environments during early life may incur costs, simply because they lack the stimulation necessary for optimal development [10,11]. There have been several studies showing that rearing environments that allow a greater expression of natural behavior are beneficial for later success in the adult environment [12]. For example, rearing chicks with access to perches during the first weeks of life or rearing them in aviaries compared to cages can have positive and long-term effects on spatial ability and learning [10,13]. Regarding stress coping ability and immune responses, having access to litter and perches during rearing can buffer against different stressors, both in laying hens [14,15] and quail [16]. Such studies indicate that adding resources to a barren environment, thereby making it more complex for young birds in their early life, has both immediate and long-lasting effects on the modulation of their coping phenotypes, as well as on their immunocompetence. Nevertheless, these enriched commercial environments for laying hens are still considerably less complex than the natural environment in which their ancestors evolved.

As illustrated above, most studies investigating the effect of the early environment on poultry welfare, behavior, and physiology focused on a comparison between barren and enriched early environments. It would be, nevertheless, interesting to deepen our understanding of how early environmental complexity can affect birds’ development, and specifically their ability to cope with different challenges. Furthermore, as was pointed out by Campbell et al. [12], there is a knowledge gap regarding the link between (and mechanisms behind) complex rearing environments and immunocompetence. Environmental complexity can be increased by offering different resources (which is the usual approach and is mentioned in the studies above), but it can also be increased by offering different forms of the same resource. Given the close connections between choice and controllability [17] and between controllability and coping responses [18], it is possible that it is the experience of making choices that is the underlying reason for the positive effects found from rearing in complex environments.

This study focused on the effects of providing chicks with increased possibilities to express choice as a form of environmental complexity. More specifically, we aimed to elucidate the effects on coping-related traits in laying hen chicks, by offering increased variation within two resources, i.e., perches and litter, known to be important during early rearing (reviewed by Janczak and Riber [19]). By providing litter and perches in both treatments, our single-choice environment corresponded to the complex environments that were used in previous studies [14,20]. Our multi-choice environment then implied a further increase in complexity, by increasing the possibility to express choice within these important resource types. Variables representative of fear, learning, and stress responses, as well as variables for exploring the immune status were selected. We hypothesized that this increased complexity would promote both behavioral and physiological coping abilities in chicks. In practice, this would mean that they are better prepared for challenges, as well as opportunities, in commercial poultry production settings.

## 2. Material and Methods

### 2.1. Animals and Husbandry

Day-old laying hen chicks (females) of the white hybrid Bovans Robust were purchased from a Swedish commercial hatchery (Swedfarm AB). After the standard procedure of handling and sorting at the hatchery, chicks were transported 255 km in a temperature-controlled vehicle to the Swedish Livestock Research Centre. On arrival, all 104 birds were weighed and placed in one of eight pens in the same room at an indoor facility. The birds were randomly assigned to each pen, but they were checked so there were no significant differences between pens in the average weight per bird (average weight per bird was 36.2 ± 0.13 g). Each pen was 1.2 × 2.4 m and housed 13 birds. This low stocking density, compared to commercial conditions, was chosen so that all chicks could potentially use a particular litter box or perch at the same time. The birds were provided with water and feed (commercial standard) ad libitum throughout the entire study, and these were checked and refilled as necessary every morning. The average light intensity in the pens was 20.2 ± 1.67 lux. There was a heat lamp hanging above the middle of the pen during the whole experiment. The average temperature was 43.6 ± 1.65 °C under the heat lamps and 23.6 ± 0.26 °C in the rest of the pen. All birds were marked with numbered leg rings at two weeks of age. At the end of the experiment, when birds were 22-day-old, they were weighed.

### 2.2. Treatments

Before chicks arrived, pens were assigned to one of two treatments, according to a balanced design. These treatments differed in their level of complexity and this was achieved by varying the types of the key resources: the perches and litter material provided in the pen. The perch types were round rubber, braided rope, flat wood, and flat wire. The litter types were straw, wood shavings, sand, and peat.

**Figure 1:**
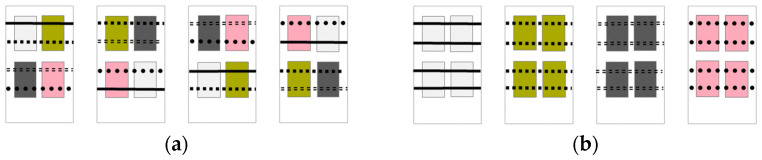
Overview of the treatments: (**a**) multi-choice, with all four types of litter and perches presented in each pen, and (**b**) single-choice, with only one type of litter and perch type per pen. The location of each litter and perch type was balanced across the four multi-choice pens, to control for potential effects of position within the pen on chick usage. Furthermore, each litter and perch type in the multi-choice pens was represented in a single-choice pen. In this way, we could account for any potential effects of specific perch and litter types, since our aim was not to explore the effects of the different litters and perches themselves, but the effects of increased choice per se.

In one treatment, all four litter and perch types were offered in each pen (4 pens in total, Figure 1a). This treatment was consequently named the “multi-choice treatment”. Test litters were presented in four trays (71 × 35 × 3.5 cm). To prevent location bias within the pen, litter and perches were placed such that the position of the different types of resources was not repeated between pens. In the other treatment, only one litter type and one perch type were offered in each pen (4 pens in total, Figure 1b). This treatment was subsequently named the “single-choice treatment”.

Each of the litter and perch types presented in the multi-choice environments was presented in one single-choice environment. In this way we could account for potential effects of specific perch and litter types, since our aim was not to explore the effects of the different litters and perches themselves, but the effects of increased choice in the offered resources per se. The pairings were as follows: wood shavings–rope, sand–wood, straw–rubber, and peat–wire. Perch height started at 15 cm and was elevated to 45 cm at 14 days of age. Pen walls were covered to minimize visibility between neighboring pens.

### 2.3. Physiological Measures

Based on previous studies [14,16,21,22], four variables were investigated to assess the status of each individual’s immune system: (1) The lymphoproliferative response to phytohaemagglutinin-p (PHA-P), a cellular representative of the immune system reflecting birds’ pro-inflammatory potential; (2) Interferon gamma (IFN-Ɣ) plasmatic concentration, as a pro-inflammatory mediator; (3) Natural antibodies against sheep red blood cells (SRBC), reflecting general humoral immune capacity; and (4) Heterophil/Lymphocyte (H/L) ratio, a representative of cellular immunity widely used as a hematological indicator of underlying chronic stress responses. The sampling procedure lasted two days and started when chicks were 16 days old. The lymphoproliferative response required an intradermal injection and an in vivo analysis the day after, while the other three variables were analyzed in vitro with blood sampled on one occasion. Blood was sampled 24 h post lymphoproliferative induction. All chicks were sampled on the same day, to account for any potential carry-over effects for the procedure.

For the lymphoproliferative or swelling response to PHA-P, a 0.05 mL solution of PHA-P (*Phaseolus vulgaris* lectin from Sigma Aldrich; Saint Louis, USA) in phosphate buffer saline (PBS), 1 mg/1 mL solution, was injected into the left wing web of each chick, according to previous descriptions [14,22]. After 24 h (±1.5 h), the thickness of the pre-marked injection site was measured and compared with basal thickness, measured just before the injection. The thickness was measured using a digital caliper (Cocraft^®^) with an accuracy of 0.03 mm. The indicator of swelling was obtained using the following calculation: percentage of swelling = (basal thickness/thickness post 24 h) × 100 [16]. For accuracy and intra-observer reliability, measures were repeated in order to obtain two measures that differed by less than 5%, after which an average of these was used.

For blood sampling, a maximum of 0.75 mL was obtained from the right brachial vein of each chick (opposite wing from the PHA-P response induction). Syringes were prepared with anticoagulant ethylenediaminetetraacetic acid (EDTA). Blood smears were made immediately, using one drop from the syringe according to standard practice, while the remaining blood was placed on ice in a transport box. Blood was then centrifuged at 2000 rpm for 10 min to obtain plasma, which was stored at −20 °C until further analyses. IFN-Ɣ was quantified using a validated species-specific ELISA kit (Ray Bio^®^ Chicken IFN-gamma ELISA Kit, ELG-IFNg). The minimum detectable dose was assessed to be 0.06 ng/mL. Procedures specified by the manufacturer were followed, and the concentrations for all chicks were determined the same day and on the same plate. The intra-assay coefficient of variation (CV) was 5.91%. Natural antibodies (Nab) against SRBC were assessed using a microagglutination assay [23]. Procedures were similar to those conducted for investigating acquired antibody responses for SRBC. An amount of 25 µL complement-inactivated (through a thermal bath at 56 °C) plasma was serially diluted in 25 µL of PBS (1:2, 1:4, 1:8 up to 1:512). Then, 50 µL of a 2% suspension of SRBC in PBS was added to the wells. Microplates were covered with aluminum foil, incubated at 40 °C for 1 h and checked for agglutination every 15 min. Hemagglutination of the test plasma samples was compared to the blanks (PBS only) and negative controls (wells with no SRBC suspension). The same person conducted the analysis of all the samples with an inter-assay CV of 9% and an intra-assay CV of 7%. Antibody titers were reported as the Log_2_ of the highest dilution yielding significant agglutination. Blood smears were stained with May Grünwald Giemsa, and differential counts of 100 white cells per smear were made, according to previous practice [14,21]. All counts were made by the same person with an intra-plate CV of 2.1%. The H/L ratio was then calculated by dividing the number of heterophils by the number of lymphocytes. The same person obtained all blood samples, and the same person held chicks for blood withdrawal. Blood collection took less than 90 s for each chick.

### 2.4. Behavioral Measures

The treatment effects on behavior were evaluated using two behavioral tests when the chicks were three weeks of age: one on an individual level, a tonic immobility (TI) test; and one on a pen level, a repeated opportunity test. This time interval was chosen to give chicks time to recover from the stress associated with the physiological measurements.

The duration of TI response is thought to reflect an individual’s level of fearfulness [24] and is frequently used in poultry research. TI was induced by placing a chick on its back and then a hand was lightly held against its chest for 15 s. If the individual moved within three seconds, induction was repeated a maximum of three times. The number of attempts to induce TI, latency to first head movement, latency to first vocalization, and latency to standing up from the tonic position (TI duration) were registered. Individuals who were not induced after three attempts were given a TI duration of one second, while individuals not standing up after five minutes received the maximum score of 300 s. Three different people conducted the test according to standardized procedures, so that all chicks could be tested on the same day within a period of five hours. Treatments were balanced between different test operators. Before the test started, inter- and intra-observer reliability were secured by joint evaluations of test chicks, so that the CV was <10% for all latency measurements.

**Figure 2:**
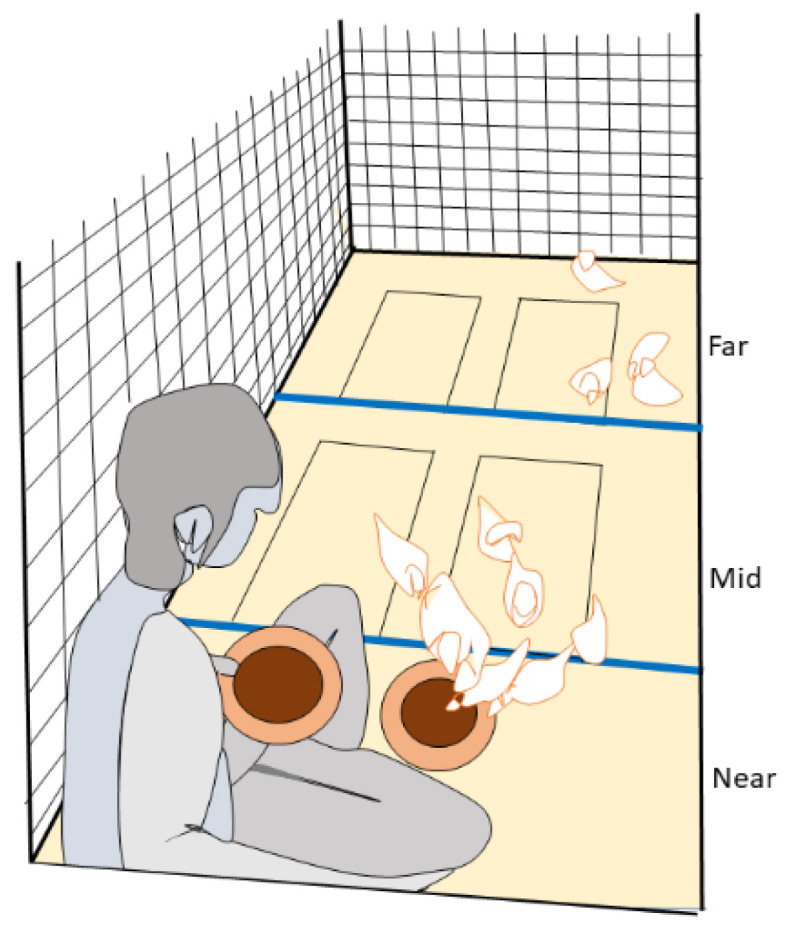
Schematic view of a pen during the repeated opportunity test. Each pen was divided into three areas: “far”, “mid”, and “near”. During the second phase (the last three repetitions), a tester sat down with legs crossed in the pen, placing one feed bowl on the ground and one in her lap, as illustrated.

The repeated opportunity test was constructed to explore the ability of chicks to adapt to routine procedures, i.e., repeated exposure to an initially novel situation, as well as ability to take opportunities, i.e., access to an attractive food source. The test consisted of two phases, with an increased challenge level in the second phase. Each phase consisted of three repetitions, and all were carried out in each pen. In the first phase of the test (repetitions 1–3), the test operator opened the entrance door to the pen and placed an initially novel object (a porcelain bowl) with initially novel feed (ten live mealworms) mixed with an initially novel litter (crushed straw pellets) in the home pen for 90 s. The bowl was placed in the middle of the pen, one arm’s length from the entrance, before closing the door. Time and video recording started once the door was closed. In the second phase (repetitions 4–6), the challenge level was increased by the test operator actually entering the pen and sitting down in the corner by the entrance. She presented two feed bowls (the same bowls and content as in the first three repetitions) for 90 s (see Figure 2). One bowl was placed on the ground in front of her, in a similar position to in phase 1, whereas the other bowl was held on her lap. She had her gaze downwards and to the right, avoiding eye contact with the chicks. An assistant closed the pen door while the tester sat down. Time and video recording started once the door was closed. There was at least one hour between each repetition.

This test can be considered as a series of challenges that the chicks have repeated opportunities to overcome, in order to access the food reward. Given that the chicks were initially allowed to move to the far end of the pen, the latencies for overcoming consecutive challenges can be placed in the following order: latency to approach the mid and then the near part of the pen (Figure 2), latency to peck in the bowl on the ground (repetitions 1–6), latency to jump up onto the person, and, finally, latency to peck in the bowl on the person’s lap (repetitions 4–6). The latency recorded was the time for the first chick in the pen to overcome the challenge. Additionally, the number of chicks in each area of the pen (far, mid, near) every 10 s and the total number of pecks to each bowl were determined. The total number of worms eaten in each bowl was registered by counting the number of worms remaining in the bowl at the end of the test, as an indicator of overall success. The test can therefore reflect coping, as well as exploration and learning. All measures, apart from counting the number of worms, were made by the same observer from the video recordings. Before the analysis, an intra-observer reliability of CV < 10% was secured for all behavior variables.

### 2.5. Statistical Analysis

Statistical analyses were conducted in R software (version 3.3.2; Development Core Team, 2016). Means, calculated using mixed models, are presented as estimated marginal means, and error values show the standard errors of this estimated mean. Linear mixed models, fit using the restricted maximum likelihood (REML) and lme4-package, were used for variables, showing a normal distribution and homoscedasticity. Significant fixed effects were investigated using Type III ANOVA, with Kenward–Roger approximation of degrees of freedom, and the lmer Test package.

For all mixed models, pen ID was used as a random effect, to account for potential influences between chicks in the same pen and to control for the effect of having different types of litter and perches in each pen (see Figure 1), while treatment (“single-choice” and “multi-choice”) was used as a fixed effect. This approach allowed us to move from comparing different types of litter and perches to focusing instead on the statistical effects of different levels of environmental complexity. These models were fit for “swelling response to PHA-P”, “weight increase”, and “latency to first head movement (TI)”. If the random effect was too small, resulting in convergence issues, this type of model was dropped, and a general linear model was fit instead. This was the case for the variables “H/L ratio” and “latency to stand up (TI)”. For variables showing non-normality and/or heteroscedasticity, average values per pen were explored for treatment effects using Kruskal–Wallis tests. This was the case for the variables “natural antibody titers”, “IFN-Ɣ plasmatic concentrations”, and “number of attempts (TI)”. The results for these variables are presented as means and standard errors.

In the linear mixed models investigating variables from the repeated opportunity test, interactions between “treatment”, “phase”, and “repetition” were included as fixed effects. Since the aim of this test was to explore treatment differences in the ability to improve with each repetition through learning or adaptation, pairwise comparisons of repetition differences were explored within each phase within treatments, by stratifying repetitions by phase and treatment. This comparison was always made if the effect of “repetition”, “phase”, or their interaction was marginally significant *p* ≤ 0.1 in the ANOVA. The potential risk of false positives was taken into account using the Tukey method. The Tukey method was used to adjust *p*-values and control for multiple comparisons in all linear models. Transformed values are presented as back-transformed, apart from the natural antibody titers.

For integrating information from all the variables with individual chick data, a multivariate approach was used (MVN package). A lineal discriminant analysis was used, with the different treatments as a priori categories. The different rearing environments “multi-choice” vs. “single-choice” were considered as the different classes in this analysis. In this way, within-class distance was minimized and the between-class distance was simultaneously maximized, to achieve the maximum class discrimination. The used variables (standardized before analysis) were “swelling response to PHA-P”, “natural antibody titers against SRBC”, “H/L ratio”, “IFN-Ɣ plasmatic concentrations”, “latency to stand up”, and “number of attempts to induce the TI state” in the TI test. A dispersion graph (biplot) was constructed, to visualize both the experimental individuals and the variables in the same space.

## 3. Results

One chick from a “multi-choice” environment was euthanized during the first week of the experiment because of a leg injury. Furthermore, blood withdrawal was not successful for all chicks, resulting in a lower number of individuals used in the immunology-related analyses. Exact numbers for each analysis are given in the legend to the figure. There was no difference between treatments in the overall weight gain of chicks (“multi-choice”: 182 ± 3.85 g vs. “single-choice”: 184 ± 3.85 g; F_1,6_ = 0.12, *p* = 0.73).

### 3.1. Immunological Treatment Effects

**Figure 3:**
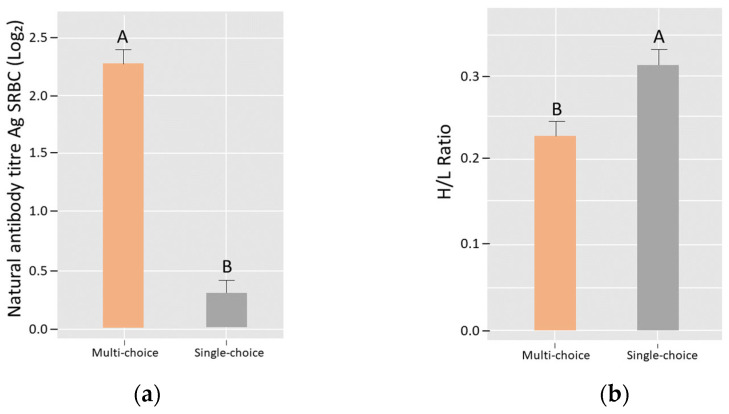
Immunological effects of “multi-choice” and “single-choice” treatments for (**a**) natural antibody titer against sheep red blood cells (SRBC), presented as the Log_2_ of the highest dilution yielding significant agglutination (mean and SE; “multi-choice”: 39; “single-choice”: 40 chicks were analyzed) and (**b**) H/L ratio (estimated marginal mean and SEM; “multi-choice”: 36; “single-choice”: 39 chicks were analyzed) in blood sampled from 16-day-old domestic fowl layer chicks. Different letters (A,B) indicate significant treatment differences.

A main effect of treatment was found for natural antibodies against SRBC, where chicks reared in a “multi-choice” environment had higher natural antibody titers than their counterparts reared in “single-choice” environments (χ = 5.33, df = 1, *p* = 0.02; Figure 3a). A treatment effect was also found for H/L ratios, where chicks reared in “multi-choice” environments had lower H/L ratios compared to chicks from “single-choice” environments (F_1,74_ = 6.92, *p* = 0.01; Figure 3b). No effect of the treatment was found on the inflammatory response to PHA-P (“multi-choice” = 94.2 ± 3.03; “single-choice” = 95.3 ± 3.03; F_1,6_ = 0.06, *p* = 0.81), nor on the IFN-Ɣ plasmatic concentration (“multi-choice” = 10.96 ± 1.84; “single-choice” = 11.13 ± 2.18; χ^2^ = 0.08, df = 1, *p* = 0.77).

### 3.2. Tonic Immobility Test

Compared to chicks from “single-choice” environments, chicks from “multi-choice” environments required more attempts to induce the TI state (“multi-choice” = 1.14 ± 0.03; “single-choice” = 1.02 ± 0.02; χ^2^ = 4.57, df = 1, *p* = 0.03) and they had a shorter latency to standing up after TI had been induced (“multi-choice” = 68.1 ± 10.6; “single-choice” = 109.5 ± 17.0; F = _4.67_, df = 1, *p* = 0.03). No treatment differences were found regarding latency to first head movement (“multi-choice” = 37.35 ± 9.06; “single-choice” = 37.78 ± 9.16; F_1,6_ = 0.001, *p* = 0.97) or first vocalization (“multi-choice” = 39.14 ± 3.86; “single-choice” = 37.39 ± 10.20; χ^2^ = 0.19, df = 1, *p* = 0.66).

### 3.3. Repeated Opportunity Test

**Figure 4:**
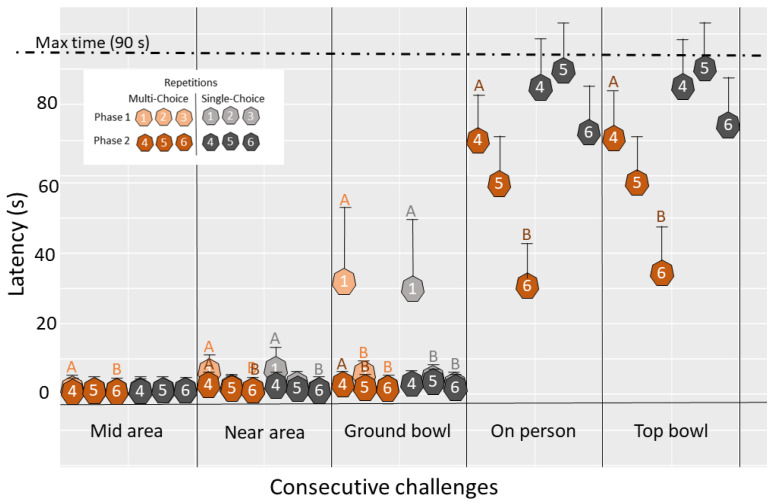
The approach dynamics in the repeated opportunity test. Dot plot (estimated marginal mean and SEM) showing the approach dynamics with each repetition during the repeated opportunity test of chicks from “multi-choice” and “single-choice” treatments. The first phase (repetitions 1–3) involved one food bowl on the ground, while the second phase (repetitions 4–6) involved a person inside the pen, one food bowl on the ground and another in the lap of the person. Latencies to overcome the challenges are shown in seconds, to enter the mid area, to enter the near area, to peck in the ground food bowl, to jump up on the person where the top food bowl was located, and, finally, latency to peck in the top food bowl. Different letters (A,B; note also the different shades within each color) indicate significant differences between repetitions, i.e., improvements with repetition, within each treatment and the different phases of the test.

During the first phase of the repeated opportunity test (repetitions 1–3), chicks from both treatments showed significant reductions in their latency to reach the mid (“multi-choice”: t = 2.45, df = 30, *p* = 0.05) or near area of the pen (“multi-choice: t = 2.73, df = 30, *p* =0.03; “single-choice”: t = 3.96, df = 30, *p* = 0.001) and in their latencies to start pecking in the ground bowl (“multi-choice”: t = 4.83, df = 30, *p* ≤ 0.001; “single-choice”: t = 4.22, df = 30, *p* ≤ 0.001; Figure 4). In the second phase (repetitions 4–6), only chicks from the “multi-choice” environment showed improvements in the consecutive challenges shown in Figure 4. Significant reductions in latencies for “multi-choice” chicks in this phase were found for latency to reach the near area (t = 3.96, df = 30, *p* = 0.001), start pecking in the ground bowl (t = 2.91, df = 30, *p* = 0.02), jump up on the person (t = 2.76, df = 30, *p* = 0.04), and to start pecking the top bowl (t = 2.77, df = 30, *p* = 0.04). 

**Figure 5:**
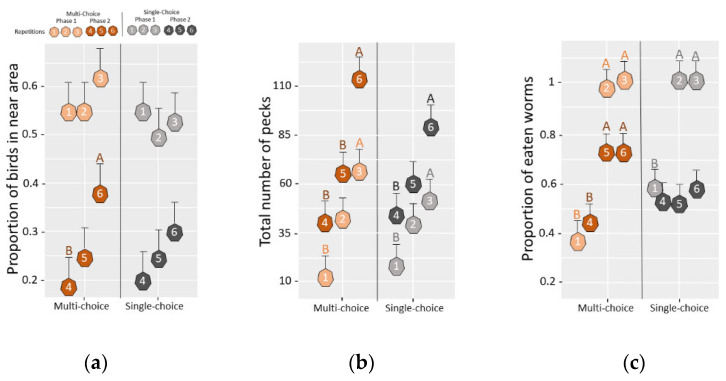
The response dynamics in the repeated opportunity test. Dot plot (estimated marginal mean and SEM) showing the response dynamics with each repetition during the repeated opportunity test for chicks from the “multi-choice” and “single-choice” treatments, regarding (**a**) the proportion of chicks located in the near area (the area where the ground food bowl was located for repetitions 1–3 and where both food bowls and the test person were located in repetitions 4–6), (**b**) the total number of pecks to feed bowl(s), and (**c**) the proportion of worms eaten during each repetition. Different letters (A,B; note also the different shades within each color) indicate significant differences between repetitions within treatments and phases.

Compared to the first phase, and irrespective of treatment, pens in the second phase of the test (when the experimenter was in the pen), had a lower average proportion of chicks in the near area (0.55 ± 0.04 vs. 0.25 ± 0.04; F_1,30_ = 100.37, *p* ≤ 0.001; Figure 5a), a higher average number of pecks (68.4 ± 6.39 vs. 38.2 ± 6.39; F_1,30_ = 30.2, *p* ≤ 0.001; Figure 5b) and a lower average proportion of eaten worms (0.58 ± 0.03 vs. 0.82 ± 0.04; F_1,27_ = 29.62, *p* ≤ 0.001; Figure 5c). In pairwise comparisons investigating how chicks from each treatment improved with repetition, there was no increase in the proportion of chicks in the area near the novel bowl in the first phase (*p* > 0.05). However, in the second phase in the “multi-choice” environments, there was a significant increase in the proportion of chicks in the near area between the fourth and sixth repetition (t = −2.47, df = 30, *p* = 0.049). No equivalent increase was found for chicks from “single-choice” environments (Figure 5a). Chicks from both environments showed an increase in the number of pecks with repetition in both phases (*p* ≤ 0.05; Figure 5b) and in the proportion of worms eaten in the first phase (*p* ≤ 0.05; Figure 5c). However, in the second phase, there was only a significant increase in the proportion of worms eaten for “multi-choice” environments (t = −2.78, df = 30, *p* = 0.025; Figure 5c).

### 3.4. Multivariate Treatment Effects

**Figure 6:**
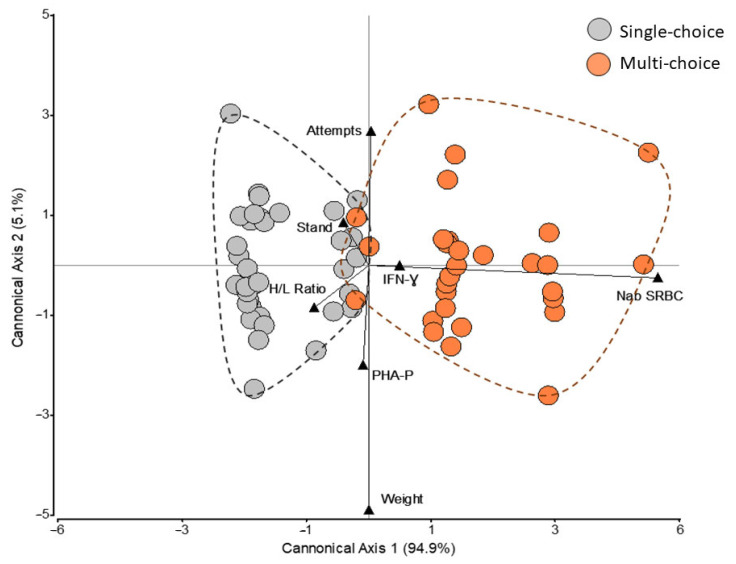
Exploration of the discriminatory capacity of the two treatments. Lineal discriminant analysis, including the following standardized variables (shown in black triangles): inflammatory response against phytohaemagglutinin-p (PHA-P), natural antibody titer against sheep red blood cells (Nab SRBC), heterophil/lymphocyte (H/L) ratio, IFN-Ɣ plasmatic concentrations (IFN-Ɣ), latency to stand up in a tonic immobility test (Stand), and number of attempts to induce the TI state (Attempts) and weight gain (Weight). Each dot represents a laying hen chick in the study for which the register of all variables was complete. Grey dots represent chicks reared in the “single-choice” environment (37 chicks were analyzed), whereas orange dots represent chicks reared in the “multi-choice” environment (32 chicks were analyzed).

Figure 6 shows a lineal discriminant analysis using those variables measured individually for chicks in this study: inflammatory response against PHA-P, Nab production against SRBC, H/L ratio, IFN-Ɣ plasmatic concentrations, weight gain, latency to stand up, and number of attempts to induce the tonic state in a TI test. The two treatments are defined by the distribution of the colored dots in the discriminant space determined by the canonical axes. The figure shows an effective discrimination of the individuals according to their a priori treatment: being reared in “multi-choice” or “single-choice” environments. This discrimination can be clearly observed in canonical axis 1 (94.9% of variability between the groups explained), for which natural antibody titers against SRBC and the heterphil/lymphocyte ratio are the two most important (discriminant coefficients of 0.98 and 0.20, respectively).

## 4. Discussion

Chicks reared in an environment where there was a variety of different litters and perch types showed improved immune potential, indicators of diminished fear and stress responses, as well as increased exploratory behavior compared to chicks reared in a similar environment but without variety within these resource types. The results support our hypothesis that the increased complexity, achieved by providing more choice in the environment, altered the phenotype of the chicks, by boosting their coping abilities. In practice, this better preparation for environmental challenges could be a practical way to improve chick welfare. Although cost and bird performance were early key considerations in poultry production, bird health and welfare are now also important considerations [25]. The novelty of this work lies in how we changed the complexity of the environment, which was done by offering chicks the possibility to choose between different perch types and different litter materials, while keeping the total allocation of resources the same. This allowed us to move beyond the effects of providing basic resources. Additionally, we analyzed immediate effects (those found during the first three weeks of life), whereas most previous research in the area of early environmental manipulation in domestic birds falls within one of two categories: (1) prenatal/parental and in ovo manipulations (reviewed by Dixon et al. [8]), or (2) early manipulations with effects analyzed later in life (youth or adulthood) (reviewed by Campbell et al. [12]). Maternal passive immunity protection lasts until about two weeks post hatch [26], so our variables were collected when the chick was learning to rely on its own immune system. We first discuss the results from the immune related variables that were selected as indicators of the chick’s ability to resist a potentially pathogenic challenge, using non-pathogenic techniques. We then go on to discuss the behavioral results and how they relate to a chick’s ability to learn in new and potentially challenging situations, as well as how success in such a situation may be influenced by fearfulness. Finally, we return to the broader issue of how the early environment can influence the later phenotype and how that knowledge might be advantageous when rearing commercial laying hens.

A difference was found when quantifying natural antibodies, where chicks reared in the multi-choice environments showed higher circulating concentrations. Natural antibodies are present in non-immunized individuals and cover a broad specificity repertoire [27]. They originate from continuous stimulation by exogenous microbes, or correspond to the secretion of naturally occurring auto-reactive B cells, or both [28]. It is likely that the multi-choice environment, especially due to the various litter types, could have had a more diverse microbe community, greater pathogenic load, and a wider pathogenic diversity (as previously proposed for enriched conditions [16,29]), which triggered the higher production of natural antibodies. Natural antibodies are of great importance, because they are key to activating other immunological compartments, such as the complement system and adaptive immune responses [27,30,31]. It was also this variable that had the greatest discrimination power between our treatments. The results suggest that chicks reared in an environment with various litter and perch types had the advantage of a potentially better prepared immune system compared to those with the same allocation of resources but no variation. In the long run, survival would be enhanced, based on studies in hens that proposed a relation between elevated natural antibody concentration and increased probability of surviving the laying period [30]. No treatment effect was found on the in vivo pro-inflammatory potential nor in the IFN-Ɣ concentrations, which implies chicks were equally prepared to deal with a potential pathogen requiring inflammatory milieus for its clearance [21,22]. This information gives a clue, for the first time, about the specificity of the immunological effects of increased environmental complexity. It starts to fill the gap mentioned by Campbell et al. [12], and points towards an enhanced immunological potential related to humoral mediators and the series of responses that are dependent on natural antibodies being activated. This would provide the chicks reared in the multi-choice environments with the advantage of a potentially faster activation of these responses, thus reducing the time and energy allocated to immune coping.

Regarding the behavioral variables, both the tonic immobility and the repeated opportunity test were able to identify specific treatment effects that also supported our hypothesis that offering more choice in the early environment improves the coping abilities of young chicks. Chicks reared in multi-choice environments required more attempts to induce tonic immobility and showed a shorter latency to stand up, suggesting that they were less fearful than chicks from single-choice environments [24]. Previous studies found that increased environmental complexity, by adding enrichments, can result in less fearful chicks [32]. This is advantageous from a welfare perspective, since the production environment usually contains various potential stressors that can lead to fear states. Increased fear has been found to be associated with negative consequences, such as increased feather damage; low body weight, egg weight, and feed intake; and even mortality [33], all of which may indicate impaired adaptability. The repeated opportunity test, specifically constructed and designed for testing our hypothesis, illustrated treatment differences attributable to differences in the ability of chicks to adapt to challenges associated with rewarding opportunities. The lower proportion of chicks in the area close to the feed bowls in the repetitions involving a human supports that the second phase of the test was more challenging (as it was intended to be). In the first phase, pens from both treatments improved with repetition, shown as reduced latencies to approach and peck at the food bowl and an increased proportion of worms eaten, thereby indicating increased exploration and some level of adaptation. However, in the second and more challenging phase, only chicks reared in multi-choice environments showed an improvement over repetitions in these same variables and so were better able to take the opportunity to access the additional food reward. One possible explanation for the difference in the repeated opportunity test and the greater success of chicks from the multi-choice environments could be that they had experience of approaching and using various forms of resources from day one. We have previously shown, using the same litter and perch types, that chicks prefer certain litter types for certain behaviors and that different perch types affect chicks’ ability to land on them [34]. This suggests that the multi-choice environment would have given chicks a more diverse training in the behaviors involved in perch use, such as jumping and balancing, as well as increased and diversified foraging and dustbathing opportunities. It is therefore possible that the multi-choice environments led to an improved exploration and learning ability. Learning ability in farm animals has been shown to affect adaptability to a novel environment [3]. Another possible explanation, now focusing on the lack of success to exploit a new food opportunity in the chicks reared in the single-choice environments, could refer to fear and priming. While repeating a test situation can result in decreased reaction times [35], no such improvements are seen if the repeated stimulus is experienced as too aversive [36]. That chicks from the single-choice environments were more fearful was supported by the previously mentioned results from the tonic immobility test. There is no reason to expect a difference in food motivation between chicks in the different treatments, as food was always freely available in all pens.

One could suggest that the chicks’ responses in the repeated opportunity test would be comparable to their response during routine procedures in their home pen, for example when the caretaker entered to check feed and water supplies. That chicks from the single-choice environments had a higher H/L ratio, a physiological indicator of underlying chronic stress responses, implies that the chicks from this treatment were having more difficulties in coping with these everyday situations. This is in keeping with other studies showing that birds from non- or less-enriched environments had increased circulating chronic stress mediators [12]. Laying hen chicks are physiologically ready to process stress at day one [6] and the experience in commercial hatcheries has been shown to be stressful for them [37]. The chicks in our experiment were exposed to the typical husbandry procedures, i.e., incubation, handling, post-hatch feed and water deprivation, and being subsequently transported and placed in the poultry barn. Our results could also be interpreted as suggesting that early and increased environmental choice could help to alleviate the effects associated with these routine but nevertheless challenging events, supporting the results of Campderrich et al. [14].

In the context of the adaptive developmental plasticity theory [9], the increased stimulation available to chicks in an environment offering several variants of litter and perches seems to have had both immediate and potentially long lasting positive effects. All chicks were obtained from the same hatchery and randomly allocated to treatments, thus their prenatal environment could be assumed homogenous [8]. Furthermore, the physiological and behavior tests were carried out on the same days for all the chicks, restricting the interpretation of the results to the effects of the experimental treatments themselves. The influence of each treatment led to a phenotype with particular characteristics, as shown in the discriminant analysis. Chicks from the multi-choice environments also had emergent characteristics as a group, as evidenced by the quicker changes in the behavioral variables (latency to approach and to peck in the feed bowl) and greater success in exploiting novel food sources (proportion of worms eaten) in the repeated opportunity test. The traits that were found to be more pronounced after being reared in the multi-choice environments can define a more “adaptive” phenotype, as they are associated with enhanced coping abilities for a variety of future challenges, while those traits found among chicks from the single-choice environments collectively define a “less adaptive” phenotype. Our results could represent an early expression of the silver-spoon phenomenon [38]. That is to say, “multi-choice chicks” could be considered as having the advantage of growing up in a stimulating environment, with different variants of resources to “pick and choose” between, allowing for optimal development regarding the coping abilities studied. Environmental choice can allow a greater individual fit and, in this way, increase overall welfare [39]. It is also possible that choice on its own is rewarding [17], although it may be necessary to adapt the relative proportions of the different variants of the resources to avoid competition for particularly attractive litter or perch types.

That increased choice is the most likely explanation for the results is strengthened by the fact that, even though the four different pens in the single-choice treatment each contained only one litter and perch type, together they offered the same four litter and perch types as the pens in the multi-choice treatment. If the pens in the single-choice treatment pens had all contained the same litter and perch type, then it would not have been possible to exclude that the beneficial effects of being reared in a multi-choice treatment pen were attributable to some aspect of the novel litter or perch type. Apart from supporting that chicks do seem to choose between variants [34], it is also possible that the different combinations of litter and perch types, even in the single-choice treatment, may have affected the physiological and behavioral development of the chicks. We cannot assess this, since we did not have all possible combinations of the litter and perch types. In future studies, increased replication, especially of the different options within the single-choice treatment, might make it possible to explore how different litter and perch types (alone or in combination) influence the variables measured. It would also be interesting to explore the relative benefits of the number of choice options for a specific resource (in our study the increase was from one to four options).

## 5. Conclusions

In summary, the results support our hypothesis that increased complexity, achieved by providing young chicks with several variants of resources to choose between in their environment, can potentiate both behavioral and physiological coping abilities. The immunological, stress coping, and behavioral results obtained were indicative of the laying hen chicks being better prepared for immediate, and potentially for future environmental challenges, while at the same time possessing a greater potential to adapt and thus make better use of opportunities.

## Data Availability

The datasets generated during and/or analyzed during the current study are available from the corresponding author on reasonable request.
